# A Comparative Study of Antimicrobial and Antioxidant Activities of Plant Essential Oils and Extracts as Candidate Ingredients for Edible Coatings to Control Decay in ‘Wonderful’ Pomegranate

**DOI:** 10.3390/molecules26113367

**Published:** 2021-06-02

**Authors:** Tatenda Gift Kawhena, Umezuruike Linus Opara, Olaniyi Amos Fawole

**Affiliations:** 1 Department of Horticultural Science, Faculty of AgriSciences, Stellenbosch University, Stellenbosch 7600, South Africa; 19547129@sun.ac.za; 2 SARChI Postharvest Technology Research Laboratory, Africa Institute for Postharvest Technology, Faculty of AgriSciences, Stellenbosch University, Stellenbosch 7600, South Africa; 3 UNESCO International Centre for Biotechnology, Nsukka 410001, Nigeria; 4 Postharvest Research Laboratory, Department of Botany and Plant Biotechnology, University of Johannesburg, Johannesburg 2006, South Africa

**Keywords:** alginate, chitosan, lemongrass, thyme, oregano

## Abstract

This study determined the antimicrobial and antioxidant activity of lemongrass (LO), thyme (TO), and oregano (OO) essential oils and ethanolic extracts of pomegranate peel (PPE) and grape pomace (GPE) as candidate ingredients for edible coatings. Antifungal effects against *Botrytis* *cinerea* and *Penicillium* spp. were tested using paper disc and well diffusion methods. Radical scavenging activity (RSA) was evaluated using 2,2-diphenyl-1-picrylhydrazyl and 2,2′-azinobis-3-ethylbenzothiazoline-6-sulfonic acid assays. Gas chromatography-mass spectrometry analysis identified limonene (16.59%), α-citral (27.45%), β-citral (27.43%), thymol (33.31%), paracymene (43.26%), 1,8-cineole (17.53%), and trans-caryphellene (60.84%) as major compounds of the essential oils. From both paper disc and well diffusion methods, LO recorded the widest zone of inhibition against tested microbes (*B. cinerea* and *Penicillium* spp.). The minimum inhibitory concentrations of LO against *B. cinerea* and *Penicillium* spp., were 15 µL/mL and 30 µL/mL, respectively. The highest (69.95%) and lowest (1.64%) RSA at 1 mg/mL were recorded for PPE and OO. Application of sodium alginate and chitosan-based coatings formulated with LO (15 or 30 µL/mL) completely inhibited spore germination and reduced the decay severity of ‘Wonderful’ pomegranate. Lemongrass oil proved to be a potential antifungal agent for edible coatings developed to extend shelf life of ‘Wonderful’ pomegranate.

## 1. Introduction

Decay incidence caused by pathogens affects fresh produce resulting in postharvest losses, often translating to marked economic losses on the supply chain [1,2,3]. Decay reduces food safety, sensory quality, and shortens the storage and shelf life of fresh produce [4]. Furthermore, losses due to decay limit the availability of fresh produce on the market, causing exorbitant prices, which restrict market growth [5]. Research shows that decay occurs on the supply chain of fresh horticultural produce during several phases, including transportation, cold storage and shelf life [5,6,7,8]. Several pathogens have been implicated as causal agents for postharvest decay in fresh produce, such as *Alternaria* spp., *Colletotrichum gloeosporioides*, *Botrytis cinerea*, *Penicillium expansum*, *P. sclerotiorum* and many others [5,9].

Postharvest management of disease causing pathogens in fresh produce has been predominantly done by applying fungicides [5,10]. The successful use of fungicides to reduce decay incidence has been highlighted for fruit types such as stone [11], apple [12], pear [13], mango [14] and many others. Moreover, the use of fungicides such as methyl 2-benzimidazole carbamate, thiabendazole and fludioxinil (FLU, Scholar^®^, Syngenta) to prevent decay has been widely reported in pomegranates and pome fruit [15,16,17].

For pomegranate (*Punica granatum* L.), a highly nutritional and commercially important fruit, pathogens, namely *B. cinerea* and *Penicillium* spp., were identified as the main causal agents of wound and latent infections leading to decay [8,18,19]. When the fruit is dipped in FLU before cold storage, the fungicide solution inhibits fungal spores on the calyx part of the fruit, thereby reducing microbial proliferation [15,19]. However, at present, consumer preference for fresh produce without fungicide application is increasingly growing, and several countries have imposed strict regulations on the minimum residue levels for fungicides [19]. Furthermore, studies have proven that several pathogens develop resistance to synthetic fungicides, and waste disposal of most fungicides poses a threat to the environment [20]. Therefore, studies have been initiated to investigate biodegradable treatments as alternatives to replace the synthetic fungicide application on pomegranate and other fruit types.

The use of essential oils as an alternative to synthetic fungicides has shown great potential to control postharvest diseases in fresh produce [21,22]. Essentials oils (EOs) are volatile, partially flammable, and contain aromatic compounds which are extracted by hydrodistillation or pressing processes [23]. The major components of EOs responsible for inherent antimicrobial and antioxidant properties include terpenes, polyphenols, terpenoids, quinones, flavonoids, aldehydes, fatty acids, alkaloids, and lectins [24,25,26]. Essential oils such as lemongrass (LO), thyme (TO), carvacrol, clove, cinnamon and oregano (OO) have the “generally regarded as safe” (GRAS) status and, therefore, can be used for fresh produce preservation [27]. Traditionally, EOs have been used for flavouring of food products, alternative medicine, sanitation, phytotherapy, and food preservation [25,26,28]. For example, a study reported by Ali et al. [29] proved that LO (28 µL·L^−1^) vapour reduced the development of anthracnose on Papaya (*Carica papaya* L.) during nine days of storage at ambient temperature (25 °C). Most EOs reduce microbial proliferation by directly lowering mycelial growth and spore germination by minimizing cellular metabolism [21]. In addition, hydrophobic EOs reportedly reduce microbial growth by disrupting cell membrane integrity and promoting electrolyte leakage leading to cell death [30]. Despite numerous studies showing EOs as a biodegradable postharvest treatment to control decay in stone and pome fruit, few studies have been extended to study the effect of EOs on other commercially important fruit types, including pomegranate.

Non-volatile plant extracts (PEs) derived from materials such as pomegranate peel, grape pomace, garlic and ginger, are also an alternative to synthetic fungicides used to exert antimicrobial effects against pathogenic microorganisms on fresh produce/food products [31,32,33]. These products are extracted from plants using solvents, microwave, and ultrasonic assisted methods, and are further purified for use as antimicrobial agents on fresh produce [34]. The major components of PEs includes compounds such as quercetin, gallic acid, kaempferol, and flavan-3-ols catechin, which are responsible for antimicrobial effects against fungi (*B. cinerea* and *Penicillium digitatum*) [33,34]. Successful application of PEs to control decay has been reported for citrus [33], sweet cherries [35], papaya [31], mango [36], and many others. For instance, Nicosia et al. (35) reported that application of pomegranate peel extract (PPE)(1.2 and 12 g/L) on artificially inoculated sweet cherries and lemon reduced the incidence of postharvest rots caused by fungi (*B. cinerea* and *Penicillium* spp.) during storage at room temperature (22–24 °C). Similar to EOs, research work on the application of PEs to extend storage and the life of pomegranate fruit is still limited. 

When EOs and PEs are applied on fresh produce or food products to enrich postharvest treatments such as edible coatings, modified atmospheric packaging, controlled atmosphere storage, refrigeration and irradiation, studies have reported the maintenance of microbial and sensory quality [37,38,39]. In particular, the incorporation of EOs and PEs into edible coatings (ECs) widens their functionality to become carriers of active compounds and has been found to reduce microbial proliferation [39]. Furthermore, novel active packaging technologies developed from biodegradable ECs formulated with EOs and PEs extended storage and shelf life of pomegranate arils [40], avocado [41], banana and papaya [31,42], plum and orange [43,44], amongst others. Moreover, studies reported by Gull et al. [45] showed that pre-storage application of nanochitosan (1%) coating solutions containing PPE (1%) significantly reduced microbial proliferation in ‘Rival’ apricot fruit which minimized the decay incidence during 30 days of cold storage (4 °C). Despite the widespread application of EOs and PEs on food products, a few challenges have been reported in their application. When applied in large amounts, the odour of EOs often intensifies, which may negatively alter the final sensory quality of food products [46,47]. Likewise, the incorporation of high concentration of PEs in ECs may result in off-flavours. Perumalla and Hettiarachchy [48] identified several factors affecting the selection of PEs for use in ECs, including cost effectiveness, final sensory quality of food product, availability, and bioactive properties. Cerqueira et al. [49] outlined that, while higher concentration of PEs is associated with greater antioxidant capacity, the active components of PEs may negatively alter permeability, thermal, and mechanical properties of the ECs. 

The antimicrobial properties of EOs and PEs have been documented extensively [50,51]. Furthermore, studies have explained the mode of action of several EOs and PEs with different constituent compounds [52]. From such studies, several authors have derived the optimal concentration of EOs and PEs for the formulation of ECs from the minimum inhibitory concentration (MIC) values for a broad spectrum of pathogens [44,53]. The MIC is often assigned to the smallest concentration of EO or PE, which prevents the growth of a test microorganism [24]. This technique is often effective to optimize the concentration of active components of ECs for the best quality of the final food product. However, differences have been noted in MIC values in studies conducted for the same EOs and/or PEs. For instance, the MIC value reported for LO (*Cymbopogon citratus*) against *Escherichia coli* was 0.25%, which was higher than 0.12% reported by Naik et al. [54] and Guerreiro et al. [47]. Both Faleiro [55] and Hyldgaard [56] attributed the difference in test strains and methodology used as major factors affecting MIC values observed. In addition, EOs and PEs often have different aromatic compounds even when they are extracted from the same plant [46]. Therefore, in developing novel ECs for fresh food preservation, it is important to determine the optimal concentration of candidate bioactive ingredients before the formulation process. 

Considering the low residue levels set for fungicides in many export destinations, and the importance of antimicrobial coatings as a biodegradable alternative, the current study was carried out to determine the antioxidant activities of the candidate EOs and PEs for formulating novel ECs, and to test antimicrobial activity against important disease pathogens affecting pomegranate (*Punica granatum* L.) fruit.

## 2. Results

### 2.1. Chemical Composition of Essential Oils

The chemical composition of the lemongrass (LO), thyme (TO), and oregano (OO) are presented in Table 1. Based on GC–MS analysis, 57 volatile compounds (VOCs) belonging to nine different chemical classes were identified. For LO, the most abundant VOs were monoterpenoids (88.97%), followed by alcohols (8.02%), sesquiterpenes (1.5%), ketones (0.56%) and aldehydes (0.14%). The major compounds identified for LO were α-citral (27.45%), β-citral (27.43%), and limonene (16.59%). Thyme oil consisted of monoterpenes (91.67%), sesquiterpenes (0.09%), alcohols (4.88%), aldehydes (0.44%), and phenylpropene (2.05%). The major compounds identified for TO were thymol (33.31%) and para cymene (43.26%). Oregano oil had VOCs including sesquiterpenes (65.59%), monoterpene (29.26%), sesquiterpenoids (0.55%), monoterpenoids (4.85%), ketones (0.1%), and phenylpropene (0.1%). The compound trans-caryophyllene (60.84%) and 1,8-cineole (17.53%) contributed the largest percentage of the chemical composition of OO.

### 2.2. In Vitro Antifungal Properties

#### 2.2.1. Disc and Well Diffusion Tests

**Figure 1:**
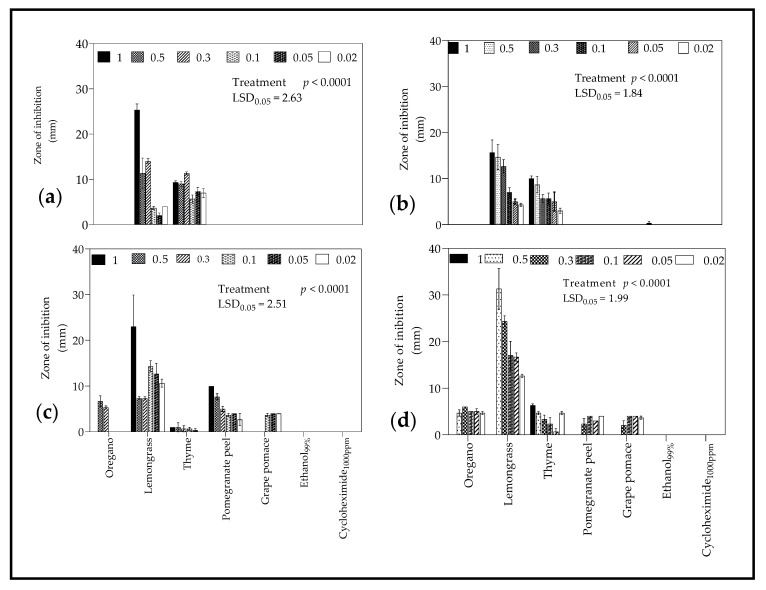
Zone of inhibition (mm) of *Botrytis cinerea* (**a**,**c**) and *Penicillium* spp. (**b**,**d**) as determined by disc (**a** and **b**) and well (**c**,**d**) diffusion test methods in plates treated with different concentrations (0.02–1 µL/mL) of essential oils and plant extracts. Plates were inoculated with a spore suspension (1 × 10^9^ spores·L^−1^) of the test organism (*B. cinerea* and *Penicillium* spp.) Ethanol (99.9%) and cycloheximide (1000 ppm) were used as controls. Vertical bars represent the standard error (SE) of mean values of three replicates (1 plate = replicate). LSD_0.05_ represent least significant difference (*p* < 0.05).

The disc and well diffusion test results for *B. cinerea* and *Penicillium* spp. are summarized in Figure 1. For the test fungi, the diameter of the ZI were relatively wider when observed using agar well diffusion test than the disc diffusion test. In addition, disc diffusion test showed LO and TO as the most effective EOs for both fungi; whereas, OO showed no inhibition effect. However, for the same EOs, the agar well diffusion test showed some differences compared to the disc diffusion test. For *B. cinerea*, using the disc diffusion test (Figure 1a), LO had the widest ZI (*p* < 0.0001), at an average of 25.33 mm (1 µL/mL), followed by TO (11.33 mm; 0.3 µL/mL) and others. Similarly, from well diffusion test (Figure 1c), LO had the widest ZI (*p* < 0.0001) of 23.00 mm (1 µL/mL), followed by PPE (10 mm; 1 µL/mL), OO (6.67 µL/mL; 0.5 µL/mL), TO (3 mm; 0.5 µL/mL) and others. Likewise, for *Penicillium* spp. using the disc diffusion test (Figure 1b), LO had the widest ZI (*p* < 0.0001) of 15.67 mm (1 µL/mL) followed by TO (10.00 mm; 1 µL/mL) and others. Using well diffusion test (Figure 1d), LO recorded the widest ZI of 31.33 mm (1 µL/mL), and followed by TO (6.33 mm; 1 µL/mL), OO (4.66 mm; 0.5 µL/mL) and others.

#### 2.2.2. Minimum Inhibitory Concentration against *Botrytis cinerea* and *Penicillium* spp.

**Figure 2:**
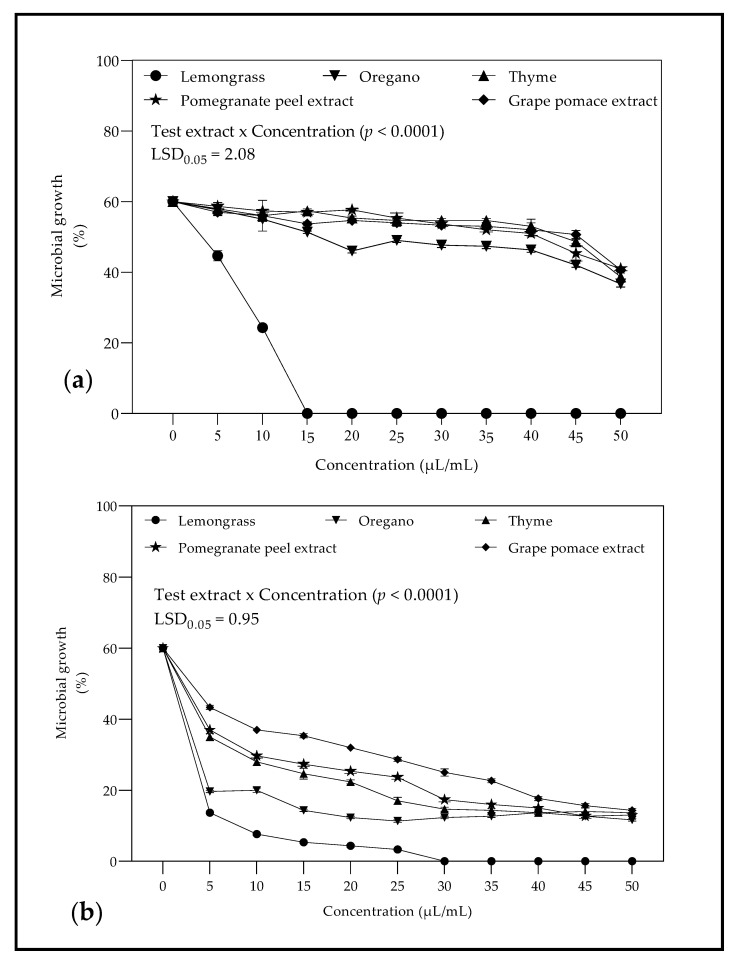
Antifungal activity (µL/mL) of essential oils and plants extracts indicating the minimum inhibitory concentration against (**a**) *Botrytis cinerea* and (**b**) *Penicillium* spp. Vertical bars represent the standard error (SE) of mean values of three replicates (1 plate = replicate). LSD_0.05_ represent the least significant difference (*p* < 0.05).

Initial screening of EOs and PEs using disc and well diffusion test identified LO as the most active EO at the different concentrations (0.02–1 µL/mL) tested against *B. cinerea* (Figure 2a) and *Penicillium* sp. (Figure 2b), LO proved to be the most active EOs with a minimum inhibitory concentration (MIC) of 15 µL/mL and 30 µL/mL for *B. cinerea* and *Penicillium* spp., respectively. 

### 2.3. Radical Scavenging Activity

**Figure 3:**
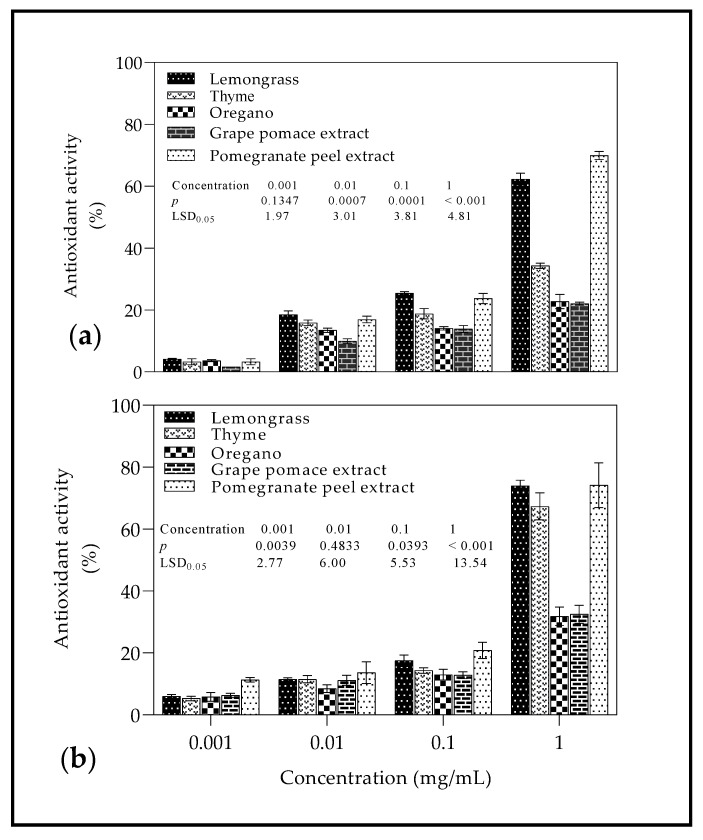
Antioxidant capacity of essential oils and plant extracts at different concentrations (0.1–1 mg/mL) evaluated by (**a**) DPPH and (**b**) ABTS assays. Vertical bars represent the standard error (SE) of mean values of three replicates. LSD_0.05_ represent least significant difference (*p* < 0.05).

Using DPPH method (Figure 3a) at different concentrations (0.1–1 mg/mL) of EOs and PEs, radical scavenging activity (RSA) was in the range 1.64–69.95%. There was an increase in RSA as the concentration of EOs and PEs was increased, with PPE and GPE recording the highest (69.95%) and lowest (22.07%) RSA at 1 mg/mL, respectively. For ABTS method (Figure 3b), a similar trend was observed with RSA in the range 5.33–74.17%, PPE and OO recording the highest (74.95%) and lowest (31.83%) RSA at 1 mg/mL, respectively.

### 2.4. In Vitro and In Vivo Investigations with Edible Coatings

#### 2.4.1. Spore Germination

**Figure 4:**
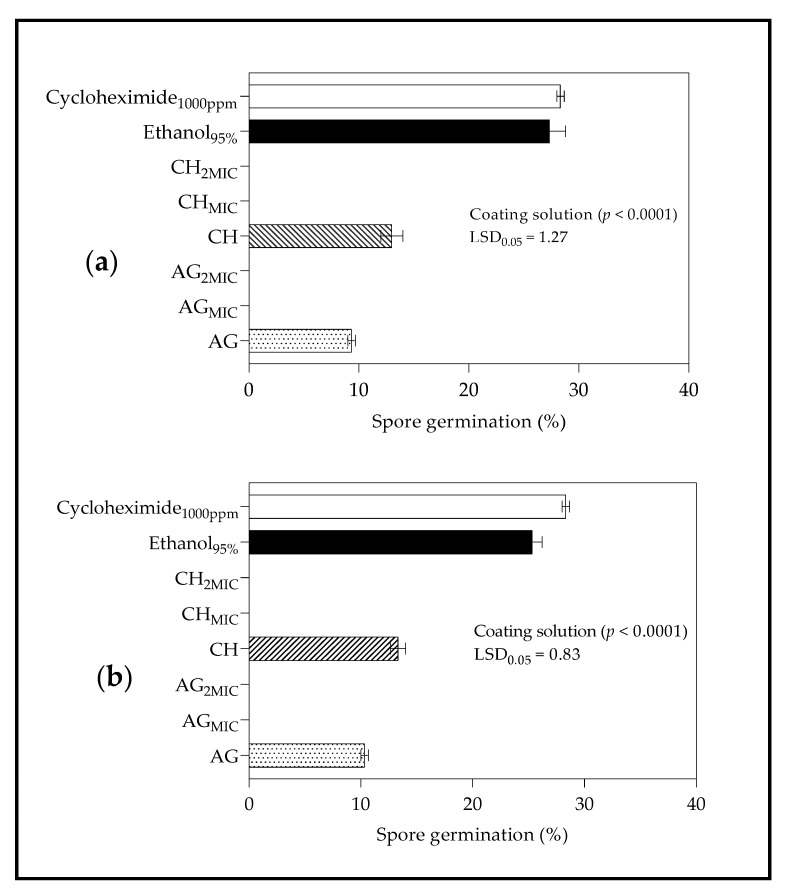
Effect of edible coating solutions on spore germination (%) of (**a**) *Botrytis cinerea* (**b**) *Penicillium* spp. after 24 h of incubation at 22 °C (*B. cinerea*) or 25 °C (*Penicillium* sp.). MIC—minimum inhibitory concentration; 2MIC—double the minimum inhibitory concentration. *B. cinerea* (MIC = 15 µL/mL; 2MIC = 30 µL/mL) and *Penicillium* spp. (MIC = 30 µL/mL; 2MIC = 60 µL/mL); CH—chitosan; CH_MIC_—chitosan formulated with lemongrass oil at minimum inhibitory concentration (15 or 30 µL/mL); CH_2MIC_—chitosan formulated with lemongrass oil at double the minimum inhibitory concentration (30 or 60 µL/mL); AG—sodium alginate; AG_MIC_—sodium alginate formulated with lemongrass oil at the minimum inhibitory concentration (15 or 30 µL/mL); AG_2MIC_—sodium alginate formulated with lemongrass oil at double the minimum inhibitory concentration (30 or 60 µL/mL). Vertical bars represent the standard error (SE) of mean values of three replicates (1 plate = replicate). LSD_0.05_ represent least significant difference (*p* < 0.05).

The results for spore germination are shown in Figure 4. For *B. cinerea*, application of coating treatments AG_MIC_, AG_2MIC_, CH_MIC,_ and CH_2MIC_ resulted in no spore germination compared to AG and CH (Figure 4a). Sodium alginate coating without LO showed lower spore germination than CH and, therefore, AG exerted a greater inhibition in spore germination than CH. Similarly, for *Penicillum* spp., coating treatments AG_MIC_, AG_2MIC_, CH_MIC_ and CH_2MIC_ showed the greatest antimicrobial activity, with no spore germination recorded (Figure 4b). Likewise, AG coating inhibited spore germination more compared to CH.

#### 2.4.2. Zone of Inhibition

**Figure 5:**
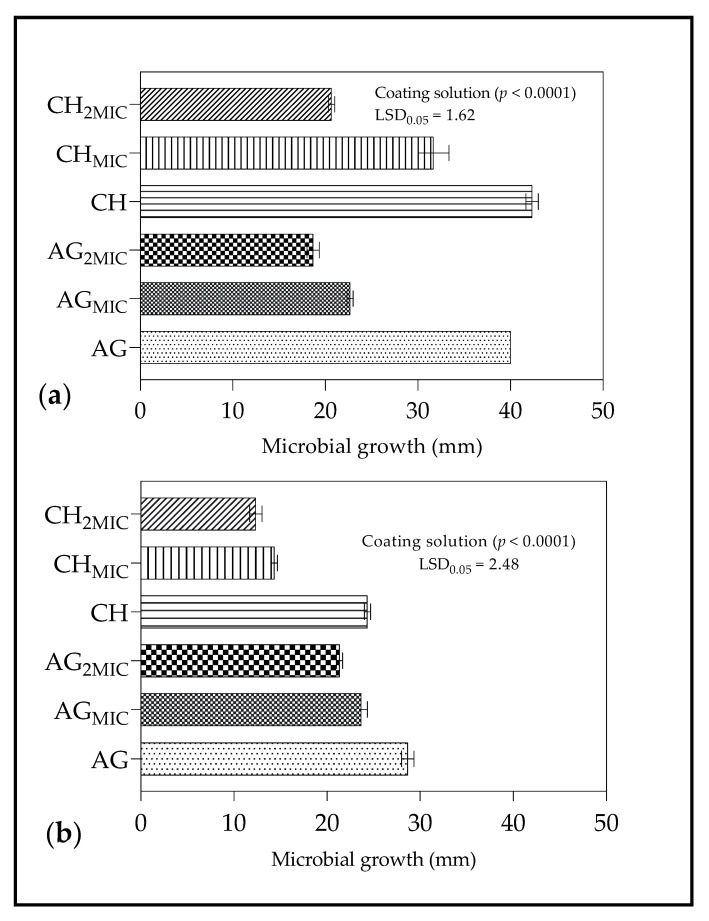
Effect of coating solutions on zone of inhibition (mm) produced after inoculation with spore suspension (1 × 10^9^ spores·L^−1^) of (**a**) *Botrytis cinerea* and (**b**) *Penicillium* spp; MIC—minimum inhibitory concentration; 2MIC—double the minimum inhibitory concentration. *B. cinerea* (MIC = 15 µL/mL; 2MIC = 30 µL/mL) and *Penicillium* spp. (MIC = 30 µL/mL; 2MIC = 60 µL/mL); CH—chitosan; CH_MIC_—chitosan formulated with lemongrass oil at minimum inhibitory concentration (15 or 30 µL/mL); CH_2MIC_—chitosan formulated with lemongrass oil at double the minimum inhibitory concentration (30 or 60 µL/mL); AG—sodium alginate; AG_MIC_—sodium alginate formulated with lemongrass oil at minimum inhibitory concentration (15 or 30 µL/mL); AG_2MIC_- sodium alginate formulated with lemongrass oil at double the minimum inhibitory concentration (30 or 60 µL/mL). Vertical bars represent the standard error (SE) of mean values of three replicates (1 plate = replicate). LSD_0.05_ represent least significant difference (*p* < 0.05).

The results for the ZI of *B. cinerea* and *Penicillium* spp. growth after application of coating treatments are shown in Figure 5. For *B. cinerea*, the order for inhibition of microbial growth was as follows, AG_2MIC_ > CH_2MIC_ > AG_MIC_ > CH_MIC_ > CH > AG (Figure 5a). For *Penicillum* spp., the order for inhibition of microbial growth was CH_2MIC_ > CH_MIC_ > AG_2MIC_ > AG_MIC_ > CH > AG (Figure 5b).

#### 2.4.3. In Vivo Antimicrobial Properties of Chitosan and Sodium Alginate Based Coatings from Lemongrass Oil 

**Figure 6:**
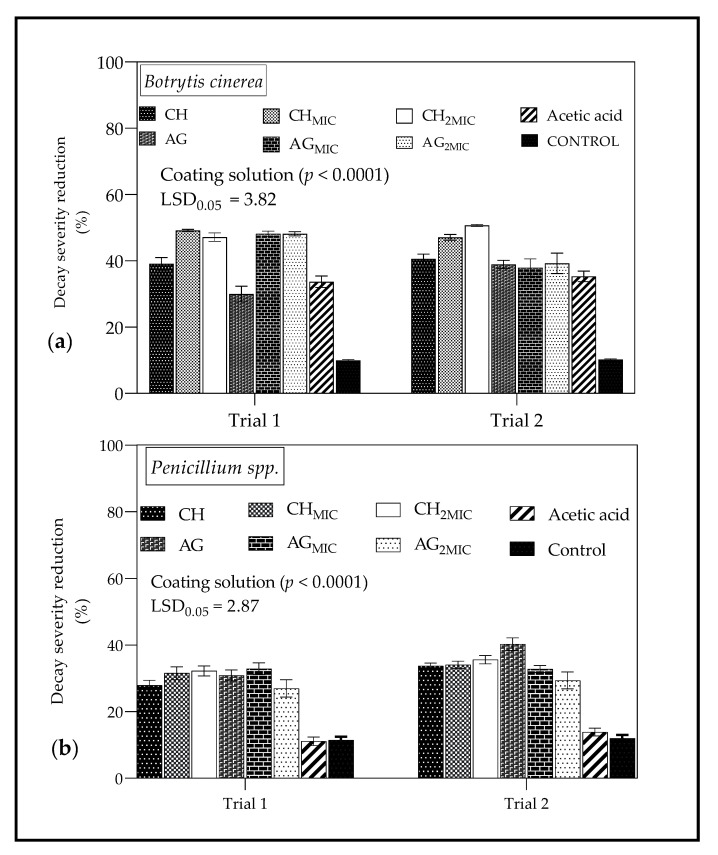
Decay severity reduction with respect to control (%) of coated ‘Wonderful’ pomegranate inoculated with (**a**) *Botrytis cinerea* and (**b**) *Penicillium* spp. and incubated for 10 day at 22 °C and 25 °C, respectively. MIC—minimum inhibitory concentration; 2MIC—double the minimum inhibitory concentration. *B. cinerea* (MIC = 15 µL/mL; 2MIC = 30 µL/mL) and *Penicillium* spp. (MIC = 30 µL/mL; 2MIC = 60 µL/mL); CH—chitosan; CH_MIC_—chitosan formulated with lemongrass oil at minimum inhibitory concentration (15 or 30 µL/mL); CH_2MIC_—chitosan formulated with lemongrass oil at double the minimum inhibitory concentration (30 or 60 µL/mL); AG—sodium alginate; AG_MIC_—sodium alginate formulated with lemongrass oil at minimum inhibitory concentration (15 or 30 µL/mL); AG_2MIC_—sodium alginate formulated with lemongrass oil at double the minimum inhibitory concentration (30 or 60 µL/mL). Vertical bars represent the standard error (SE) of mean values of three replicates (5 fruit = replicate). LSD_0.05_ represent least significant difference (*p* < 0.05).

Based on the in vitro results (Figure 2) for LO, the MIC against *B. Cinerea* was 15 µL/mL (1.5% *v*/*v*) and 30 µL/mL (3% *v*/*v*) against *Penicillium* spp. These concentrations were selected for the formulation of CH and AG coatings for in vivo experiments. The results of the in vivo study of antimicrobial properties of coatings formulated from LO are shown in Figure 6. Moreover, Table 2 shows images of coated ‘Wonderful’ pomegranates inoculated with both *B. cinerea* and *Penicillum* spp. after 10 days of storage. As shown in Figure 6, application of coating solutions reduced the decay severity caused by test microbes (*B*. *cinerea* and *Penicillium* sp.). For *B. cinerea* (Figure 6a), incorporation of either MIC (15 µL/mL) or 2MIC (30 µL/mL) of LO into coating solutions (CH and AG) resulted in less decay severity than uncoated fruit, particularly in the first trial (Trial 1). There were no significant differences in decay severity for fruit coated with both CH and AG coatings formulated with either 15 or 30 µL/mL of LO (Trial 1 and 2). For *Penicillium* spp. (Figure 6b), in trial 1 and 2, the incorporation of LO at a concentration of either MIC (30 µL/mL) or 2MIC (60 µL/mL) into AG and CH coatings resulted in lower decay severity compared to untreated fruit. In trial 2, for CH coatings, the difference between fruit with/without LO was not significant.

## 3. Discussion

### 3.1. Composition of Essential Oils

The chemical composition of EOs has been extensively studied. The results for chemical composition of LO resembles the study reported by Munhuweyi et al. [57], who similarly found alcohols, monoterpenoids, ketones, and aldehydes in the profile. For TO, the results corroborate with Borugă et al. [58] who identified thymol, gamma-terpinene, and para-cymene as the major components, as previously reported by Grigore et al. [59]. The results from this study agreed with Tavakoli et al. [60] who found carvacrol (30.73%), thymol (18.81%), para-cymene (10.88%), caryophyllene (7.73%), and 3-carene (4.06%) as the main components of OO. These compounds are classified under sesquiterpenes, monoterpene, sesquiterpenoids, monoterpenoids, ketones and phenylpropene as observed in this current study. However, difference were observed in the composition of OO from the study reported by Quiroga et al. [61] who found γ-terpinene (32.10%), α-terpinene (15.10%), p-cymene (8.00%) and thymol (8.00%) as the major components. The differences observed in the composition of EOs from the previous related can be ascribed to several factors including the extraction process, part of the plant used for extraction, seasonal variation, and other factors [62].

### 3.2. In Vitro Antifungal Properties

#### 3.2.1. Disc and Well Diffusion Test

The agar well and disc diffusion tests are two of the most common techniques used to determine the antimicrobial activity of plants or microbial extracts [63]. Overall, wider zones of inhibition (ZI) were observed when using agar well diffusion test than disc diffusion test. Furthermore, there were difference in ZI observed between the two methods for the same EOs/PEs for the fungi tested. From the methodology, the volume of EOs/PEs used to fill agar wells (100 µL) was larger than the volume applied to paper discs (20 µL). Therefore, we speculate that for the agar well method, there was high diffusion potential due to the volume of EOs/PEs in contact with test fungi, which may have increased the surface area to exert antifungal effects. Studies have highlighted the demerits of disc diffusion test including the inaccurate testing of slow growing micro-organisms due to the loss of microbial extracts by evaporation [64]. Thus, it may be necessary to employ both methods for accurate detection of the antifungal effects of the EOs/PEs against the test fungi.

The results showed that EOs exhibited higher antifungal activity than PEs against the fungi (*B. cinerea* and *Penicillium* spp.) tested for both disc and well diffusion test. Lemongrass oil showed the greatest antifungal activity followed by either OO or TO. The study reported by Tzortzakis and Economakis [65] found that LO (25–500 ppm) inhibited fungal sporulation and spore germination of the examined pathogens including *B. cinerea*. Similarly, studies by Guynot et al. [66] demonstrated the potential of LO against several fungi, including *Penicillium* spp. In a comparative study, LO and cinnamon oil exhibited the strongest in vitro antifungal activity of all EO_S_ tested with an MIC of 15 µL against *B. cinerea* [67]. However, the study reported by Reang et al. [68] identified TO as the EO with the maximum growth inhibition of *B. cinerea* when compared to clove, lavender, lemongrass, and peppermint. The LO used in this study had over 55% citral content (Table 1), lower than 69% than reported by Tzortzakis and Economakis [65]. This suggests that the antifungal activity of LO is a combined effect of all aromatic compounds and not only the main component citral as confirmed by Ma-In et al. [69].

#### 3.2.2. Minimum Inhibitory Concentration against *B. cinerea* and *Penicillium* spp.

The results corroborate with Siripornvisal et al. [67] who found the in vitro MIC of LO as 15 µL/mL when tested against *B. cinerea*. However, for *Penicillium* spp., the results were not in accordance with Li et al. [70], who observed the MIC of LO as 20 µL/mL, which was lower than 30 µL/mL found in this study. Several factors contribute to the difference in MIC values, which include the major components of EOs, extraction method and difference in fungi strain being tested [25]. 

### 3.3. Radical Scavenging Activity

The remarkable antioxidant properties of PPE have been reported in several studies [71,72,73]. Pomegranate peel extract is rich in polyphenolic compounds, which accounts for the inherent natural antioxidant property, which may translate into biological actions such as antimicrobial activity, anti-tyrosinase activity, and others [72,74,75]. The polyphenolic compounds in PPE include anthocyanins, catechins, flavonoids, punicalin, pedunculagin, punicalagin, ellagic acid, and many others [72,74]. The antioxidant activity of OO is often related to aromatic compounds such as rosmarinic acid, 1,8-cineole, carvacrol, and others [61,76,77]. Contrary to this study, Kulišić et al. [77] observed the strongest antioxidant activity as determined by DPPH radical scavenging method for OO compared to EOs derived from thyme (*Thymus vulgaris* L.) and wild thyme (*Thymus serpyllum* L.). The observed difference in RSA from previous studies can be attributed to the different chemical composition of EOs and extraction techniques used for extracts [78].

### 3.4. In Vitro and In Vivo Investigations with Edible Coatings

#### 3.4.1. Spore Germination

Antifungal activity against *B. cinerea* and *Penicillium* spp. demonstrated by treatments AG_MIC_, AG_2MIC_, CH_MIC_, and CH_2MIC_ can be explained by the inherent antimicrobial properties of both LO and CH [39,79]. Munhuweyi et al. [80] found that in vivo and in vitro, chitosan could inhibit the growth of *B. cinerea* and decay incidence by 18 to 66%. Similarly, research work demonstrated the efficacy of LO (10 and 50 g·L^−1^) incorporated into CH to completely inhibit the growth of *B. cinerea* and *Penicillium* spp. of pomegranate fruit [57]. During spore germination, new wall layers are formed between the original wall and the cytoplasm of fungi to promote the formation of new spores [81]. However, the application of LO disrupts cell membrane integrity leading to cell death, therefore, minimizing spore germination [30]. The antifungal effect of CH are well documented [82], however, the similar effect of CH and AG coatings on spore germination for the test fungi (*B. cinerea* and *Penicillium* spp.) suggests that AG also exhibits antimicrobial effects, previously observed when tested against Gram negative bacteria [83].

#### 3.4.2. Zone of Inhibition

The results showed that coating treatments formulated with LO recorded the widest ZI against tested microorganisms (*B. cinerea* and *Penicillium* spp.), in particular, treatments with double the MIC (CH_2MIC_ and AG_2MIC_). Lemongrass oil incorporated in AG and CH produced antimicrobial coatings which exhibited antifungal properties, as reported in previous studies [84,85,86]. Alginate and chitosan-based coatings formulated with LO have been used to maintain microbial quality for pomegranate [57], fresh-cut pineapple [85], fresh-cut apples [84], and others. 

#### 3.4.3. In Vivo Antimicrobial Properties of Chitosan and Sodium Alginate Based Coatings from Lemongrass Oil

The coating solutions formulated with LO showed the greater reduction in decay severity against test strains (*B. cinerea* and *Penicillium* spp.) than other coatings without LO. This was more pronounced in trials 1 and 2 for *B. cinerea.* Furthermore, for *B. cinerea*, doubling the concentration of LO (15 to 30 µL/mL) incorporated into CH and AG coatings resulted in lower decay severity than other coated and uncoated fruit. Azarakhsh et al. [85] showed that LO (0.1–0.5% *v*/*v*) incorporated into an alginate-based (0.3% and 0.5% *w*/*v*) coatings significantly reduced yeast and mould counts and total plate counts of coated samples compared to uncoated fresh-cut pineapple. Likewise, researchers have shown a reduction in microbial proliferation when LO-based coatings are applied on fresh-cut apples [87], fresh-cut melon [88], and others. In contrast, for *Penicillium* spp., the overall decay severity reduction by CH and AG coating treatments was lower than *B. cinerea*. This was further supported by higher MIC value (30 µL/mL) for *Penicillium* spp. than *B. cinerea* (MIC = 15 µL/mL). We speculate that *Penicillium* spp. may exhibit more resistance to antifungal effects of the coating solutions prepared in this study than *B. cinerea.*

## 4. Materials and Methods

### 4.1. Fruit Supply

‘Wonderful’ pomegranate fruit harvested at commercial maturity were procured from Sonlia Pack-house (33°34′851″ S, 19°00′360″ E), Western Cape, South Africa. Upon arrival at the Postharvest Technology Research Laboratory, Stellenbosch University, pomegranates were sanitized according to the procedure reported by Munhuweyi et al. [57] with slight modifications. Briefly, fruit were washed in milli–Q water and drenched two times in ethanol solution (700 g·L^−1^) for 1 min and once in sodium hypochlorite (3.5 g·L^−1^) for 2 min. Fruit were allowed to dry in an environmental test chamber (MedKem Laboratories, East Rand, South Africa) set at room temperature (23 °C).

### 4.2. Preparation of Plant Extracts

#### 4.2.1. Pomegranate Peel Extract

Pomegranates were manually peeled with sterilized knives (Sigma-Aldrich, Johannesburg, South Africa), and the peels were collected at −80 °C in airtight containers for further use. The fruit peels were cut into small pieces before being oven-dried in an oven dryer (Model nr. 072160, Prolab Instruments, Sep Sci., Cape Town, South Africa) operated at an air velocity of 1.0 m/s, parallel to the drying surface of peels at 50 °C until reaching a no weight change [89]. Ethanolic extracts were prepared according to Fawole et al. [72] with modifications. Briefly, ground powder (2 g) was firstly extracted in 10 mL of ethanol (80% *v*/*v*) and afterwards in distilled water (10 mL) for 1 h using an ultrasonic bath (Separation Scientific, Cape Town, South Africa) (700 W, 40 kHz and 25 L capacity). Subsequently, the solution was centrifuged for 10 min in a refrigerated (4 °C) centrifuge (Sigma 3–18 K, Osterode, Germany) operating at 4000 rpm. The extract was subjected to a rotary evaporator (G3 Heidolph, Schwabach, Germany) (40 °C) under low pressure to remove the solvent. Following that, extracts were filtered under vacuum through Whatman No.1 filter paper at room temperature for 24 h, and the filtrate was collected.

#### 4.2.2. Grape Pomace Extract

Grape pomace extract (GPE) was prepared according to Mendoza et al. [90] with some modifications. Briefly, 300 g of ground grape pomace were extracted with 1 L of ethanol (70% *v*/*v*) for 4 h using an ultrasonic bath. The solvent was recovered within a period of 3 h using a rotary evaporator (40 °C) under low pressure. To concentrate the extract, 20 mL of mill-Q water was added to the solution at 1 h intervals and the resultant extract was collected and stored at −20 °C.

### 4.3. Targeted Microorganisms

Previous studies reported by Munhuweyi et al. [91] identified *Botrytis cinerea* and *Penicillium* spp. as some of the major decay causing pathogens in pomegranate fruit. These microbes were isolated from diseased pomegranate fruit during cold storage (5 ± 1 °C, 95 ± 2% RH). Pathogenicity and virulence of isolated microorganism were investigated and confirmed according to the Koch’s postulates [57,91]. Fungal pathogens were grown in Potato Dextrose Agar (PDA) (Biolab, Modderfontein, South Africa) incubated at 25 °C for 8 to 14 days to obtain actively growing spores [91]. A solution of distilled water amended with Tween 20 (Sigma-Aldrich, St. Louis, MO, USA) at a concentration of 0.01 mL·L^−1^ was added to the PDA plates of each fungal colony. Finally, the resultant spores were counted using haemocytometer (Neubauer, Marienfeld-Superior, Lauda-Konigshofen, Germany) and optical microscope (Leica Wild M8 Transmitted Light Stereo Microscope, Wild Heerbrugg, Switzerland) and adjusted to a concentration of 1 × 10^9^ spores L^−1^.

### 4.4. Gas Chromatography-Mass Spectrometry Analysis of Essential Oils

Essential oils (EOs), namely lemongrass (LO) (*Cymbopogon citratus*), thyme (TO) (*Thymus vulgaris*), and oregano (OO) (*Origanum vulgare*), were purchased from Umuthi Botanicals (Western Cape, South Africa). All the EOs were stored at 4 °C before gas chromatography-mass spectrometry (GC-MS) analysis. Volatile organic compounds of EOs were determined by a GC-MS technique using an Agilent 6890N GC (Agilent, Palo Alto, CA, USA) gas chromatograph coupled with an Agilent 5975 B MS (Agilent, Palo Alto, CA, USA) mass selective detector as reported by Munhuweyi et al. [80]. The ZB-FFAP column (30 m × 0.25 mm id × 0.25 mm) part number 7HG-G009-11 was used to separate the components. During analysis, helium (constant flow rate = 1.3 mL·min^−1^) was used as a carrier gas and EOs were individually diluted in hexane (1:10,000), and following that, 1 mL of the mixture was injected into the column with a split ratio of 10:1. Settings for running the oven were; 50 °C for 3 min, then ramped up to 80 °C at the rate of 4 °C·min^−1^ for 2.5 min; and, finally ramped up to 250 °C at a rate of 10 °C·min^−1^, and held for 3 min. Compounds were identified by their retention times (RT). Mass spectra obtained for each sample was compared with the National Institute of Standards and Technology library (NIST; version 2.0). The mean (*n* = 3) percentage relative abundances of compounds based on the integrated peaks were used [92,93]. 

### 4.5. In Vitro Antifungal Properties

#### 4.5.1. Paper Disc Diffusion Test

**Figure 7:**
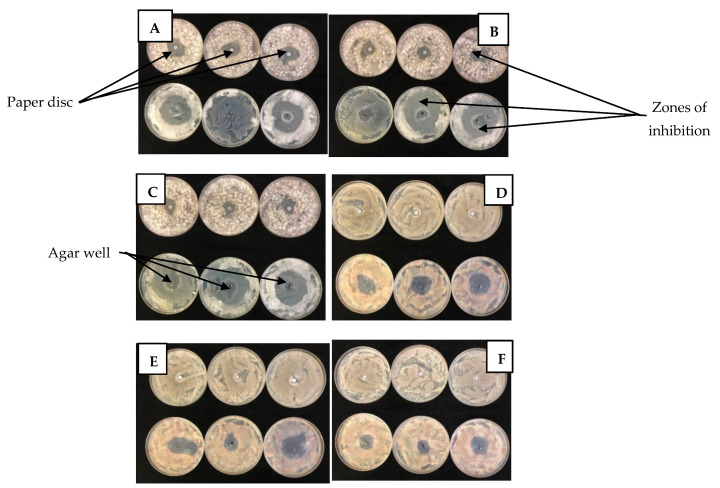
Zones of inhibition obtained by paper disc and agar well diffusion methods testing lemongrass essential oil at different concentrations ((**A**) = 1 µL/mL; (**B**) = 0.5 µL/mL; (**C**) = 0.3 µL/mL; (**D**) = 0.1 µL/mL; (**E**) = 0.05 µL/mL; (**F**) = 0.02 µL/mL) against *Botrytis cinerea* on day 4 after inoculation and incubation at 26 °C in the dark.

The paper disc diffusion test was carried out to initially screen EOs and PEs for antimicrobial activity [94]. The EOs and PEs were diluted in ethanol (99.9%) to different concentrations (0.02–1 µL/mL). Each plate was inoculated with a spore suspension (1 × 10^9^ spores·L^−1^) of the test organism (*B*. *cinerea* or *Penicillium* sp.), spread over the plate using a sterile rod and allowed to dry for 3 to 5 min (Figure 7). In triplicates, 0.6 cm discs (Munktell, Falun, Sweden) were inoculated with EOs or PEs (20 µL) and placed at the centre of the PDA (Neogen, Lancashire, UK) plate. Ethanol (99.9%) and cycloheximide (1000 ppm) were used as controls. Ethanol (99.9%) was the positive control, and cycloheximide (1000 ppm) was the negative control. Ethanol was used in diluting the EOs/PEs and was expected to offer very low antifungal effects, whereas cycloheximide is a fungicide/antibiotic produced by the bacterium *Streptomyces griseus* with known antifungal activity. Test plates were incubated at 26 °C in the dark, and the zones of inhibition (mm) were determined 4 days after inoculation.

#### 4.5.2. Agar Well Diffusion Test

Determination of antifungal activity was done according to Torres-Martínez et al. [95] with some modifications. Briefly, each plate was inoculated with a spore suspension (1 × 10^9^ spores·L^−1^) of the test organism (*B. cinerea* and *Penicillium* sp.) and spread over the plate using a sterile rod and allowed to dry for 3 to 5 min (Figure 1). The EOs and PEs were diluted in ethanol (99.9%) at different concentrations (0.02–1 µL/mL). In triplicates, holes (~0.6 cm diameter each) were cut on PDA plates using a sterile cork borer and filled with 0.1 mL of each test solution (EO or PE). Test plates were incubated at 26 °C in the dark and the zones of inhibition (mm) were measured 4 days after inoculation. Ethanol (99.9%) and cycloheximide (1000 ppm) were used as controls. 

#### 4.5.3. Determination of Minimum Inhibitory Concentration 

Minimum inhibitory concentration was identified as the lowest concentration in which the visible growth of test strains was observable [24]. Briefly, selected concentrations (0.5–50 µL/mL) of EOs were incorporated into PDA and poured into petri dishes. Agar discs (0.3 cm in diameter) covered with germinated fungal conidia served as a source of inoculums and were placed at the center of Petri dishes containing PDA with the corresponding EO. Petri dishes were sealed with parafilm to prevent the leak of test oils. The plates without EOs were used as control. The plates were incubated for 5 to 7 days and the mycelium growth (mm) was measure and expressed as a percentage of the control.

### 4.6. Radical Scavenging Activity

#### 4.6.1. 2,2-Diphenyl-1-picrylhydrazyl Assay

Determination of radical scavenging activity with 2,2-diphenyl-1-picrylhydrazyl (DPPH) assay was carried out following the procedure reported by Torres-Martínez et al. [96] with some modifications. Briefly, in triplicates, 100 µL of different concentrations (0.001, 0.01, 0.1, and 1.0 mg/mL) of EOs and PEs were mixed with 2 mL of 0.5 mmol/L DPPH in methanol. After 20 min incubation, absorbance was determined spectrophotometrically at 517 nm. The percentage of free radical-scavenging capacity was calculated by Equation (1):

Radical scavenging capacity (%) = (A_blank_ − A_sample_)/A_blank_ × 100, (1)

where A_sample_ represents the absorbance of DPPH mixed with essential oil/plant extract and A_blank_ is the absorbance of DPPH in which sample has been replaced with methanol.

#### 4.6.2. 2,2′-Azinobis-3-ethylbenzothiazoline-6-sulfonic Acid Assay

The procedure reported by Torres-Martínez et al. [96] was carried out to determine radical scavenging capacity using 2,2′-azinobis-3-ethylbenzothiazoline-6-sulfonic acid (ABTS) assay. The ABTS working solution was prepared from potassium persulfate (2.6 mM) and ABTS (2.6 mM). Briefly, in triplicates, 200 µL of ABTS working solution was added to 15 µL of test sample into wells and the absorbance was determined spectrophotometrically at 750 nm. The percentage of free radical-scavenging capacity was calculated by Equation (2),

Radical scavenging (%) = 100 − ([A_sample_ − A_blank_]/A_control_ × 100), (2)

where A_sample_ represent the absorbance of the ABTS mixed with the test sample, A_control_ represents the absorbance of the ABTS mixed with milli-Q water, and A_blank_ represents the absorbance of the sample mixed with milli-Q water.

### 4.7. In Vitro and In Vivo Investigations with Edible Coatings

#### 4.7.1. Preparation of Coating Solutions

All the coating solutions used for in vivo investigations were prepared using different concentrations of LO. The procedure reported by Gol et al. [97] was adopted to formulate chitosan coatings. One gram of water soluble chitosan (CH)(Alfa chemistry, New York, USA) powder was dissolved in 100 mL of warm milli-Q water (25 °C) and thereafter, glycerol (1% *v*/*v*) (Sigma-Aldrich, Johannesburg, South Africa), tween 80 (0.05% *v*/*v*) (Sigma-Aldrich, Johannesburg, South Africa) and LO (1.5%, 3%, 6% *v*/*v*) (Umuthi Botanicals Co., Wilderness, South Africa) were added to the solution and homogenized for 30 min in an overhead stirrer (Scientech Co., Indore, India) (2500 rpm). Sodium alginate (AG)(1% *w*/*v*) coating solutions were prepared by dissolving 1 g of sodium alginate powder (Sig-ma–Aldrich Co., Johannesburg, South Africa) in calcium chloride (2% *w*/*v*) (Sigma–Aldrich Co., Johannesburg, South Africa) solution and subsequently adding glycerol (1% *v*/*v*), tween 80 (0.05% *v*/*v*) and LO (1.5%, 3% or 6% *v*/*v*) whilst stirring using a magnetic stirrer (Spinot, Tarsons, New Delhi, India) (70 °C) [98]. Thereafter, the coating solutions were homogenized using an overhead stirrer (Scientech Co., Indore, India) (2500 rpm). 

#### 4.7.2. Spore Germination 

The effects of CH and AG coatings on *B. cinerea* and *Penicillium* spp. spore germination were determined as described by Adebayo et al. [99], with slight modifications. Briefly, in triplicates, 50 mL aliquots of the spore suspension (1 × 10^9^ spores·L^−1^) drops of test fungi (either *B. cinerea* or *Penicillium* spp.) were spread on potato dextrose agar medium supplemented with coating solutions. The plates were incubated at 22 °C for 10 to 12 h and thereafter, a drop of lactophenol cotton blue was applied to the inoculation sites on the plates to stop germination. A hand-held digital micrometer (Mitutoyo, Tokyo, Japan) under a light microscope (Olympus BX41, Tokyo, Japan) was used to determine the percentage of spore germination. Three replicate plates were used for coating solutions and a minimum of 100 spores were counted in each replicate. 

#### 4.7.3. Zone of Inhibition

To determine the zones of inhibition (ZI), 50 µL of CH and AG coating solutions were pipetted into the agar wells in plates inoculated with spore suspension (1 × 10^9^ spores·L^−1^) of test fungi (either *B. cinerea* or *Penicillium* spp.) as described by method used by Vásconez et al. [100]. After 24 h of incubation (30 °C), the inhibition zones around the wells were measured in triplicates and expressed in (mm).

#### 4.7.4. In Vivo Antimicrobial Properties of Chitosan and Sodium Alginate Based Coatings from Lemongrass Oil

The in vivo determination of antimicrobial activity of coating solutions from the selected EO was done following a method reported by Munhuweyi et al. [57] with slight modifications. Briefly, two wounds (5 mm deep by 5 mm wide) were made on the opposite sides of the sanitized pomegranate fruit. The wounds were inoculated with spore containing either 10^5^ spores/mL of *B. cinerea* or *Penicillium* spp. Inoculated fruit were then dipped into either CH or AG coating with LO (1.5%, 3% and 6% *v*/*v*). The controls were acetic acid (pH = 5, 10 g·L^−1^) with no EO and uncoated fruit. Evaluation was done on day 10 of storage (at 22 °C for *B. cinerea* and 25 °C for *Penicillium* sp.) on artificially inoculated fruit (3 replicates of 5 fruit each) and the experiment was repeated twice. Decay severity reduction (%) according to Equation (3),

Decay severity reduction (%) = [(L_f_ − L_c_)/L_c_] × 100, (3)

where L_c_ represents lesion diameter for treated fruit and L_f_ represents lesion diameter for control fruit.

### 4.8. Statistical Analysis

Statistical analysis was done using SAS Software (SAS Enterprise Guideline 7.1, Carey, NC, USA). Data were subjected to two-way analysis and the results presented as mean (±SD) values. Mean separation was performed using Fischer’s least significant difference (LSD) at 5% level of significance. Graphical presentations were made using GraphPad Prism software version 8.4.3 (GraphPad Software, Inc., San Diego, CA, USA).

## 5. Conclusions

The development of biodegradable edible coatings with antimicrobials as a control strategy for the decay and extension of shelf life of pomegranate fruit is increasingly becoming a research priority. Essential oils from lemongrass, thyme and oregano, and extracts from pomegranate peel and grape pomace exhibit promising antimicrobial effects against disease causing pathogens. The present research provides evidence of the efficacy of sodium alginate and chitosan-based bioactive coatings formulated with lemongrass oil to minimize the decay severity and as a possible postharvest control strategy. Lemongrass oil showed good antifungal effects against two major decay pathogens, *B. cinerea* and *Penicillium* spp., and when combined with either sodium alginate or chitosan on artificially inoculated pomegranates. Considering the overall results obtained, lemongrass (30 µL/mL) is recommended as an antimicrobial agent for formulating the bioactive coatings for pomegranate fruit. However, further studies to investigate the effect of lemongrass oil in different edible coatings (polysaccharides, proteins, and lipids) on fruit during cold storage and shelf life conditions are recommended.

## Figures and Tables

**Table 1:** The composition and relative abundance (%) of volatile compounds of lemongrass, thyme, and oregano essential oils determined through GC–MS analysis.

Compound	Compound	RT	Relative Abundance (%)		
			Lemongrass	Thyme	Oregano
Sesquiterpenes	α-farnesene	21.05	0.08 ± 0.01 j	-	0.39 ± 0.28 h–j
	α-ylangene	13.96	0.08 ± 0.01 j	-	-
	Caryophyllene oxide	22.04	0.06 ± 0.01 hi	-	2.75 ± 0.07 e
	γ-Cadinene	17.80	0.08 ± 0.01 j	-	0.07 ± 0.01 j
	Germacrene	17.51	0.07 ± 0.01 j	-	-
	α-Cubebene	14.15	0.16 ± 0.01 j	-	0.3657 ± 0.06 g–j
	Myrcene	7.22	0.29 ± 0.01 hij	0.09 ± 0.00 ij	
	α-Humulene	16.91	-	-	0.5888 ± 0.02 gh
	Trans-Caryophyllene	15.98	0.23 ± 0.06 j	-	60.84 ± 0.56 a
	Terpinolene	17.16	-	-	0.06 ± 0.01 j
	Cis-Caryophyllene	15.80	0.45 ± 0.01 hi	-	0.52 ± 0.05 e
Monoterpene	1,8-cineole	8.31	0.61 ± 0.04 h	2.69 ± 0.01 d	17.53 ± 0.08 b
	α-pinene	3.77	0.15 ± 0.01 ij	1.24 ± 0.01 g	4.1845 ± 0.06 cd
	Camphene	4.63	0.27 ± 0.00 ij	0.13 ± 0.03 ij	0.075 ± 0.01 j
	Limonene	8.08	16.59 ± 0.41 c	1.62 ± 0.01 f	2.21 ± 0.08 d
	Sabinene	6.05	0.05 ± 0.00 j	-	0.3680 ± 0.02 g–j
	Linalyl acetate	15.07	-	0.34 ± 0.01 hij	-
	Cymene	9.65	0.17 ± 0.01 ij	-	0.1727± 0.05 ij
	Terpinene	9.16	1.22 ± 0.03 g	-	
	α-thujone	3.86	-	0.10 ± 0.01 ij	
	Delta-3-Carene	6.78	-	0.16 ± 0.01 ij	0.2673 ± 0.00 g–j
	Exobornyl acetate	15.56	-	1.56 ± 0.03 fg	
	γ-Terpinene	9.15	-	3.26 ± 0.01 c	0.499 ± 0.00 j
	Terpinen-4-ol	15.78	-	0.32 ± 0.01 hij	
	Camphor	14.33	-	2.85 ± 0.01 d	-
	β-Pinene	5.69	0.15 ± 0.00 j	0.59 ± 0.01 h	3.9591 ± 0.04 d
Sesquiterpenoid	Isocaryophyllene	15.44	-	-	0.4734 ± 0.03 ghi
	Alpha-bourbonene	14.57	-	-	0.08± 0.00 g–j
Monoterpenoid	Citronellal	13.73	0.09 ± 0.01 ij	-	-
	Citronellol	18.13	5.03 ± 0.04 e	-	-
	α-citral	17.73	27.45 ± 0.10 a	-	-
	Geranyl acetate	18.03	1.00 ± 0.05 g	0.04 ± 0.01 j	-
	Myrtanal	12.65	0.06 ± 0.01 j	-	
	Nerol	18.59	0.32 ± 0.11 hij	0.06 ± 0.01 j	
	α-terpineol	17.17	8.32 ± 0.36 d	-	0.08 ± 0.00 j
	β-citral	17.00	27.43 ± 0.25 b	-	
	Beta-ocimene	8.95	0.06 ± 0.01 j	-	
	Thymol	23.36	-	33.31 ± 0.12 b	4.5477 ± 0.06 c
	Isocineole	7.58	-	0.09 ± 0.01 ij	
	Para cymene	9.76	-	43.26 ± 0.56 a	
	β-Phellandrene	7.21	-	-	0.22 ± 0.04 hij
	Para-cymen-8-ol	19.22	-	0.05 ± 0.01 j	
Ketones	6-methyl-5-hepten-2-one	11.12	0.31 ± 0.02 hij	-	0.066 ± 0.01 j
	4-Nonanone	10.99	0.25 ± 0.01 j	-	-
Terpene alcohol	Dihydrolinalool	14.67	0.07 ± 0.01 j	0.07 ± 0.00 ij	-
	Linalool	14.86	3.86 ± 0.02 f	3.27 ± 0.02 c	
	Cis-Linalool oxide	13.13	-	0.54 ± 0.03 h	
	Trans-Linalool oxide	13.62	-	0.33 ± 0.00 hij	-
Alcohols	1-octanol	16.70	0.06 ± 0.00 j	-	-
	Geraniol	19.24	3.87 ± 0.09 f	0.13 ± 0.01 ij	-
	Heptan-2-ol	16.72	0.16 ± 0.01 j	0.54 ± 0.02 h	
Aldehydes	Decanal	14.10	0.07 ± 0.01 j	0.44 ± 0.01 hi	-
	Octanal	16.70	0.07 ± 0.00 j	-	-
Phenylpropene	Estragole	25.18	0.36 ± 0.08 hij	2.05 ± 0.07 e	0.0873±0.01 j
		Pr > F			
Essential oil	Lemongrass oil	Thyme		Oregano	
	*p* < 0.0001	*p* < 0.0001		*p* < 0.0001	

All values are presented as mean ± SE. Means presented in the same column with different letters indicate significant differences (*p* < 0.05) according to Fischer’s least significant difference test.

**Table 2:** Decay severity of coated ‘Wonderful’ pomegranate inoculated with *Botrytis cinerea* and *Penicillium* spp. during 10 days of storage (22 °C for *B. cinerea* and 25 °C for *Penicillium* spp.).

Fungi	Treatment	Storage Duration (Days)					
		1	2	4	6	8	10
	CH	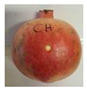	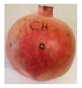	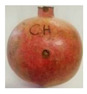	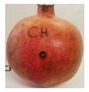	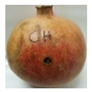	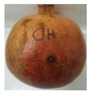
	CH_MIC_	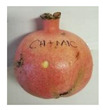	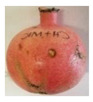	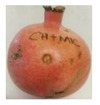	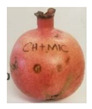	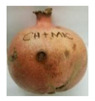	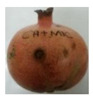
	CH_2MIC_	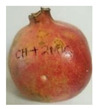	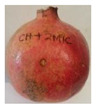	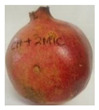	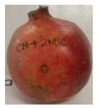	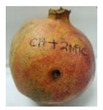	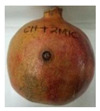
*Botrytis cinerea*	AG	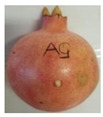	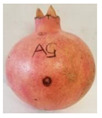	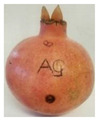	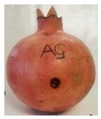	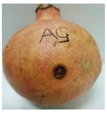	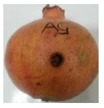
	AG_MIC_	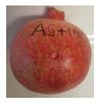	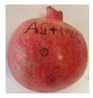	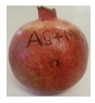	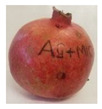	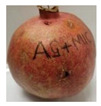	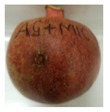
	AG_2MIC_	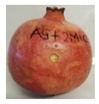	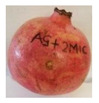	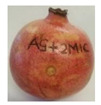	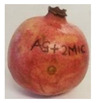	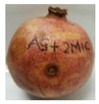	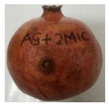
*Penicillium* sp.	CH	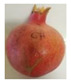	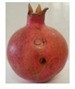	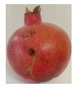	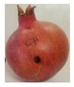	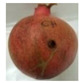	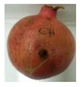
	CH_MIC_	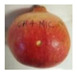	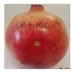	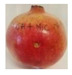	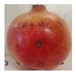	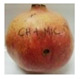	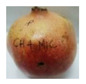
	CH_2MIC_	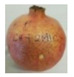	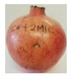	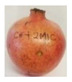	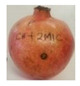	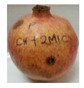	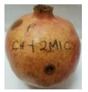
	AG	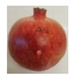	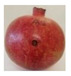	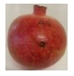	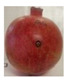	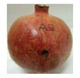	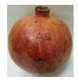
	AG_MIC_	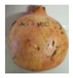	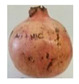	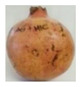	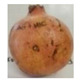	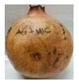	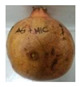
	AG_2MIC_	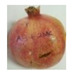	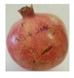	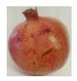	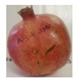	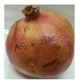	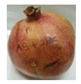
	Acetic acid	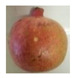	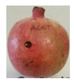	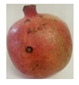	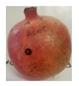	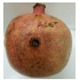	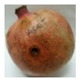
	Control	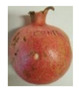	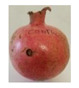	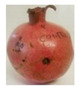	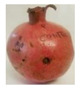	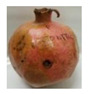	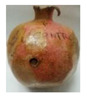

Note: CH—chitosan; CH_MIC_—chitosan formulated with lemongrass oil at 30 µL/mL; CH_2MIC—_chitosan formulated with lemongrass oil at 60 µL/mL; AG—sodium alginate; AG_MIC_—sodium alginate formulated with lemongrass oil at 30 µL/mL; AG_2MIC_—sodium alginate formulated with lemongrass oil at 60 µL/mL.

## Data Availability

Restrictions apply to the availability of these data. Data are available from the authors with the permission of funder.

## Sample Availability

Samples of the compounds are not available from the authors.
